# Supplementary material for: Uncoupling Splicing From Transcription Using Antisense Oligonucleotides Reveals a Dual Role for I Exon Donor Splice Sites in Antibody Class Switching
Source: Front Immunol. 2020 May 8;11:780. doi: 10.3389/fimmu.2020.00780 (PMC7233311; doi:10.3389/fimmu.2020.00780)
Supplement: Supplementary file 3 [file Data_Sheet_2.docx]

**SUPPLEMENTARY METHODS**

**Western blot**

Cells were lysed in radioimmunoprecipitation assay (RIPA) Lysis and Extraction Buffer (Thermo Scientific) supplemented with protease and phosphatase inhibitor cocktail. Lysates were sonicated and protein levels were quantified by Pierce™ BCA Protein Assay Kit (Thermo Scientific). Proteins were denatured at 94°C for 5 min before separation on SDS-PAGE (4–20% Mini-PROTEAN® TGX™ Precast Protein Gels were used (Bio-Rad Laboratories)). Proteins were then electro-transferred onto Trans Blot Turbo polyvinylidene fluoride membranes (Bio-Rad Laboratories). Western blots were probed with goat anti-mouse IgM Human ads-UNLB (SouthernBiotech, ref 1020-01) or goat anti-mouse IgG3 Human ads-BIOT (SouthernBiotech, ref 1100-08) antibodies. Detection was performed using an HRP-linked rabbit anti-goat secondary antibody or Extravidin-AP (Sigma-Aldrich) and chemiluminescence detection kit (ECL Plus™, GE Healthcare) using ChemiDoc™ Touch Imaging System (Bio-Rad Laboratories). Image Lab™ Software (Bio-Rad Laboratories) was used for relative quantification of the bands.
